# COVID-19 and the gendered markets of people and products: explaining inequalities in infections and deaths

**DOI:** 10.1080/02255189.2020.1824894

**Published:** 2020-10-23

**Authors:** Sarah Hawkes, Kent Buse

**Affiliations:** a Institute for Global Health, University College London, London, UK; b The George Institute for Global Health, University of New South Wales, Sydney, Australia

**Keywords:** Health policy, gender, COVID-19, inequality, intersectionality

## Abstract

COVID-19 has exposed and exploited existing inequalities in gender to drive inequities in health outcomes. Evidence illustrates the relationship between occupation, ethnicity and gender to increase risk of infection in some places. Higher death rates are seen among people also suffering from non-communicable diseases – e.g. heart disease and lung disease driven by exposure to harmful patterns of exposure to corporate products (tobacco, alcohol, ultra-processed foods), corporate by-products (e.g. outdoor air pollution) or gendered corporate processes (e.g. gendered occupational risk). The paper argues that institutional gender blindness in the health system means that underlying gender inequalities have not been taken into consideration in policies and programmatic responses to COVID-19.

## Résumé

La pandémie de COVID-19 a à la fois révélé et renforcé des inégalités de genre déjà existantes, provoquant une augmentation des inégalités dans les statistiques de santé. Les données recueillies révèlent que la combinaison de facteurs tels que l’occupation, l’ethnicité et le genre contribuent à une augmentation du risque d’infection dans certains lieux. Des taux de mortalité plus élevés ont ainsi été constatés dans des populations souffrant également de maladies non transmissibles, telles que les maladies cardiaques et pulmonaires causées par l’exposition aux effets nocifs de produits industriels (tabac, alcool, produits alimentaires ultra-transformés), de leur productions dérivées (pollution atmosphérique extérieure), ou de processus industriels genrés (risques professionnels liés au genre). Cet article démontre que le déni institutionnel des différences de genre dans le système médical a pour conséquence la non-prise en compte des inégalités de genre sous-jacentes dans la mise en place de réglementations et de programmes de réponses au COVID-19.

## Introduction

There are few positive aspects of a global pandemic, but if looking for potential moments of optimism, the global health community can take some solace in the fact that COVID-19 has served to illustrate and amplify discourse around health inequities, including the underlying structural inequalities that drive ill-health. This is not an “equal opportunities” pandemic, and the stark inequalities associated with COVID-19 have (finally) mobilised a range of voices across the media, affected communities, public health officials and politicians to confront the reasons why some parts of society have been more at risk than others. Surveillance and mortality statistics have demonstrated that the epidemic carries different levels of risk between and within societies – from risk of infection to the likelihood of disease and death. Moreover, the impact of the pandemic on the social, economic and educational wellbeing of individuals, households and communities has been similarly inequitably distributed.

For example, analysis of COVID-19 and ethnicity has served to highlight the unequal distribution of infection and risk of death. Population-based studies in the UK have found that not only are people from Black Caribbean and Pakistani ethnic groups more likely to have a positive COVID-19 test result (compared to other ethnicities), but people in the lowest economic quartile and those with the lowest levels of education also had the highest rates of infection (Niedzwiedz et al. 2020). Also, in the UK, Mulholland and Sinha (2020) reviewed evidence indicating that people from south Asian and black communities have higher rates of severe disease (likelihood of being admitted to intensive care) and higher mortality rates among health workers from these backgrounds. In Brazil, a study of the relationship between ethnicity and COVID-19 found a significantly higher risk of mortality among people from black and mixed-race ethnicities compared to white Brazilians (Baqui et al. 2020) – a result, the authors speculated, of differences in susceptibility to infection and access to health care (including specialised intensive-care services).

In many of these studies, it is the underlying social and structural determinants of the COVID-19 pandemic that are interacting and intersecting to reinforce and amplify risk of contracting the virus and subsequent poor outcomes among some ethnic groups in the population. For example, analysis by zip (postal) code of testing rates, positivity rates and proportion positive in New York City found geographical clusters of high testing rates and high positivity to be associated with ethnicity (black, Hispanic), poverty and lack of health insurance (Cordes and Castro 2020). As Selden and Berdahl note (2020), studying the distribution of COVID-19 across different ethnic groups has shone a much-needed light on the issue of “structural racism on many dimensions, including income, education, health insurance, access to medical care, access to food, health status, job characteristics, living conditions, and more”.

The United Nations Secretary General summarises these inequalities thus:

It [the pandemic] is exposing fallacies and falsehoods everywhere: The myth that we are all in the same boat. Because while we are all floating on the same sea, it’s clear that some are in superyachts while others are clinging to drifting debris. (Guterres 2020a)

In short, the COVID-19 pandemic is showing us, starkly and urgently, the importance of addressing the social and structural determinants that lie at the root of ill-health.

The remainder of this paper is devoted to understanding the role that gender, as a social and structural determinant, is playing in driving outcomes associated with COVID-19. The paper focuses predominantly on the health-related impacts of the pandemic. We do this not because we attribute a lower value to the social and economic impacts of the pandemic, but because, as professionals within the global health system, we have been closely involved in monitoring and analysing health inequalities and inequities arising from the spread of the coronavirus. Of necessity, we present empirical data to illustrate inequity, but our main focus lies in analysing why we see these inequities, what they can tell us about the underlying drivers of poor health in the twenty-first century – and, most importantly, how these inequities can illustrate where long-term change is needed to improve population health. As far as possible we take an intersectional lens to the analysis, but we recognise that there is a paucity of intersectional data on which to base much of the analysis.

## Does sex or gender drive COVID-19? In short: both

From the very first publications reporting on the emergence of a novel severe acute respiratory syndrome (SARS) corona virus – SARS-CoV-2, the virus responsible for the COVID-19 pandemic – papers published in the medical and health literature consistently pointed to the association of more severe disease and higher risk of death among men. This excess mortality was particularly noted among men in older age groups and those with pre-existing ill-health conditions (called “co-morbidities”) – more than women (Chen et al. 2020). With the privilege of hindsight, this was not surprising: similar patterns had been seen in previous coronavirus epidemics responsible for the MERS (Alghamdi et al. 2014) and SARS (Karlberg, Chong, and Lai 2004) outbreaks over the past decade.

As the global health response to a pandemic kicked into gear, and clinical case reports were supplemented by sophisticated disease surveillance data, this pattern of male susceptibility to severe infection and death has, in the main, remained constant. As part of our work as co-Directors of Global Health 50/50 (an advocacy and accountability mechanism calling for changes to the global health system to embed gender- responsiveness and gender equality within organisations and structures), we and our colleagues established a “sex-disaggregated COVID-19 tracker” – currently the world’s largest database compiling sex-disaggregated data from national (usually government) surveillance systems. We currently report on data from around 170 countries, and, in over 85 per cent of them, the risk of death is higher for men than women. On the pathway between testing, confirmation of a case and risk of death, we see higher rates of hospitalisation among men, and more severe disease (as measured by rates of admission to intensive care units) (Global Health 50/50 COVID-19 data tracker). From the 79 countries where sex-disaggregated data are available on the risk of death among confirmed cases, the risk is over one third higher in men compared to women.

The reasons why men may be more at risk of severe disease and death from COVID- 19 are many, and it is likely that no single cause can explain this health inequity. In part, biology is likely to be playing an important role. Biomedical research illustrates, for example, underlying immune system differences between men and women – differences which on the one hand appear to make men more susceptible to some infectious diseases (including viral diseases), while also making them less susceptible to autoimmune diseases compared to women (Klein and Flanagan 2016). In the case of COVID-19 in particular, there is some evidence that an enzyme called ACE-2 may be linked to higher risk of disease (Wrapp et al. 2020). ACE-2 is generally found in higher levels in men (Sama et al. 2020), and its presence may lead to a greater number of cells being susceptible to viral invasion.

Studying biomedical roots of difference may yield important insights into potential solutions for disease control. For example, identifying which enzymes or cell receptors appear to be associated with higher risks of death in men with COVID-19 infection may indicate opportunities for treatment or prevention (e.g. vaccine) interventions. However, while we appreciate the importance of a multidisciplinary approach to pandemic control that includes biological science, biology does not operate independently of the material conditions in which people live. A more “biosocial” approach to understanding health inequities highlights the ongoing interaction of sex with gender (and other social constructs) to influence and drive health inequities – and provides an opportunity to investigate how social, structural and systemic inequalities based on gender are amplifying and reinforcing any biological differences (see for example Richardson et al. 2015). As Roberts (2016) highlights, science should be investigating “the impact of social environments on human bodies … [to] show how social inequality produces disparate biological outcomes”.

## Gender as a social and structural determinant of COVID-19

“Gender” is a social construction with direct implications for how health is experienced at the level of individuals and/or embedded in the systems and institutions that drive risk of ill-health or provide treatment and care for people in the course of an illness. Driven, *inter alia,* by history, economics, cultural norms and legal systems, gender forms a basis for the division and distribution of power and resources in all societies – and thereby exerts both direct and indirect influences on health and illness across the life course (Hawkes and Buse 2020a). Gender does not act alone. Drawing on the conceptual understandings put forward by Crenshaw (1991) andhooks (1984), and proposed methodologically within Hankivsky’s work on intersectionality (2012), gender is one of multiple intersecting categories that drive experiences (including exposure to disease and access to services, for example) and outcomes, including health outcomes, across the life course.

We have previously proposed a conceptual framework for understanding the impact of gender on health outcomes (Hawkes and Buse 2020a) operating across three inter-linked axes: (i) enacted through health behaviours; (ii) embodied through intersection across social, economic and commercial determinants of health; and (iii) embedded through the institutions and systems that drive or respond to health and illness. Analysing COVID-19 across these three axes highlights the underlying roots of inequities and points to the actions needed to reduce population health risks and ensure more equitable and just health outcomes. In the next section we review the evidence for the impact of gender driving the pandemic from risk of infection to risk of death. We have identified two areas in particular where gender, intersecting with other social and structural determinants, is playing an important role in the COVID-19 pandemic: the gendered nature of occupations, including in relation to patterns of migration, and gendered patterns of exposure and consumption which lead to higher rates of population vulnerability to ill-health.

### Gender, COVID-19 and risk of infection: the impact of the gendered distribution of labour

Global data on people diagnosed as having COVID-19 infection show a relatively even distribution in terms of cases in men and women. The World Health Organization data for June 2020 found that in around 3.7 million people diagnosed as a case of COVID-19, approximately 54 per cent were men (UNWOMEN 2020); the Global Health 50/50 COVID-19 data tracker (September 2020) shows similar data in terms of global distribution of cases with slightly more cases diagnosed in men compared to women. Testing data disaggregated by sex is only available for a small number of countries, but shows that more women seek testing than men do – a finding shown by Teo et al. (2016) to be consistent with other healthcare interventions where men are less likely than women to participate in health-screening programmes. However, the global COVID-19 data hide a wide variation at both national and sub-national levels. For example, in Bahrain, Nepal, Singapore and Qatar, 80–90 per cent of people diagnosed with COVID-19 are men (GlobalHealth 50/50 data tracker), while in some countries in northern Europe (e.g. Belgium, the Netherlands, Scotland, Northern Ireland) between 60 per cent and 65 per cent of people diagnosed with COVID-19 are women.

### Can gender explain these national-level differences?

Gender experienced at the level of individuals and communities may influence factors such as who participates in screening programmes (Teo et al. 2016), and at a more structural level the gendered division of labour may also be contributing to the differences we see in the surveillance data. Gendered population demographics, intersecting with the impact of international labour migration patterns that see people from poorer countries seeking work outside their national borders, may contribute to difference in some countries. In Qatar and Bahrain, for example, 75 per cent and 63 per cent respectively of the population are male (World Bank 2020) – with a large proportion comprised of male migrant workers from south Asia and south-east Asia, and a number of low-income countries across Africa. It is estimated that there are 23 million migrant workers living in Gulf States, the majority of whom are men, and many are employed in construction and service industries (Jureidini 2014). Human rights agencies have raised concerns that the conditions under which the migrant workers are employed and housed may put them at risk of COVID-19 infection. Crowded living conditions, in particular, are likely to have contributed to the disproportionate gender imbalance in infection rates noted in both Bahrain and Qatar (Amnesty International 2020).

Nepal and Singapore have more balanced demographics (approximately equal numbers of men and women), but the former represents a sending country for large numbers of male migrants, and the latter a recipient for international labour migration. During the period 2008–2015, for example, on average around 96 per cent of the 2.7 million people issued with labour permits to leave Nepal and work overseas (in the Gulf Cooperation Countries, India and Malaysia) were men (Govt of Nepal 2016). It is thought that the recent upsurge in COVID-19 infections in Nepal has been largely driven by the return of (mainly male) migrant labourers to the country since the country began reopening in the middle of June 2020 following a strict lockdown (The Diplomat 2020). In the case of Nepal, therefore, the imbalance in reported cases among men and women may also represent biases in terms of who has access to testing: if testing is being predominantly conducted among returning migrants, then a larger number of men will be diagnosed with COVID-19 since the vast majority of labour migrants are men.

Meanwhile, in Singapore, the surge in COVID-19 infections reported among the estimated 1.2 million migrant workers in the country served to highlight the living conditions which made practices such as physical distancing and isolation extremely challenging. In a Facebook post in April 2020, the Minister of Manpower highlighted the “poor and unhygienic” living conditions under which “many foreign workers lived”, and explicitly mentioned that “To save costs, their employers would often house them at the very sites where they worked, which were unregulated” (Josephine Teo, Facebook, 6 April 2020). Such conditions proved a breeding ground for the spread of COVID-19, resulting in the higher rates of infection seen among migrant workers, most of whom are men.

Gendered rates of workforce participation along with gendered occupations may be associated with elevated risks of infection among women in some countries. Analysis of cross-national data finds that “the percent of the full-time workforce comprised by women is positively related to the percent of female Covid-19 deaths across countries” (Adams 2020, 23). Furthermore, the risks are particularly noted when working in occupations where there is increased exposure to people already infected, or the work is in close proximity and makes physical distancing very difficult. For example, where data on infection rates among health workers are available – including Germany, Italy, Spain and United States of America – the majority (70% or more) of those who are infected are women (Global Health 50/50 COVID-19 data tracker). Such figures both represent rates of exposure in higher risk environments and reflect the gendered distribution in the health workforce overall (WHO 2019a).

Globally, the majority of health and social care workers are women, frequently holding jobs in the lower quartile of pay scales in this sector (see, for example, United Kingdom Government data on gender pay gaps). Of note, people from black and minority ethnic populations in the UK are over-represented in these higher exposure occupations – including as nursing auxiliaries and assistants, occupations with the lowest median pay per hour compared to other health workers (Office for National Statistics 2020). There is some pre-liminary evidence that these higher rates of exposure among health workers are resulting in higher mortality rates too. In the British National Health Service (NHS), analysis of almost 200 deaths among health workers in the first few months of the pandemic found some indi-cation that the mortality rate for younger women was higher than that seen among non- NHS workers from the same demographic (Kursumovic, Lennane, and Cook 2020). Caution should be exercised in relation to this figure as the numbers are small and the statistic is described by the authors as “fragile”. Nonetheless, the authors do point out that since young women make up 35 per cent of the overall NHS workforce, even a small increase in death rates carries important implications for overall risk.

### COVID-19 and gendered pathways of care

The health system is not gender-neutral (Hawkes and Buse 2020b) – from the overall structure (including the financing of care), to individual patient–provider interactions,gender exerts an influence on people’s willingness and ability to engage with health systems and health-promoting programmes, including their ability to pay, and the quality of care they will receive once in the system. Among people diagnosed as a “case” of COVID-19 infection, a small proportion will go on to require hospitalisation – and in all countries where data on hospitalisation are available, the majority of those admitted are male. We have little empirical research on the impact of gender on pathways of care for COVID-19 patients, but evidence from other diseases highlights the role that gender is likely to be playing in driving health behaviours (including patterns of care-seeking) while also embedded through the programmes and practices of the entire health system.

The 2013–2015 Ebola virus (EBV) epidemic in West Africa saw a similar picture to the one described above for COVID-19: men and women had similar risks of being diagnosed with EBV infection, but men were significantly more likely to die (WHO 2016). One explanatory factor proposed by the researchers related to men’s health-seeking patterns: once symptomatic, men waited longer to seek health care compared to women – meaning that they were more infectious for longer in the community, but also that they may have been sicker once they reached hospital. Does this contribute to observed differences in COVID-19? Studies on health service use frequently confirm the relatively lower use of health services by men compared to women, but much of this is driven by women’s service use for sexual and reproductive health care needs (Wang et al. 2013). Male patterns of health-care seeking were characterised by Courtenay (2000) as “better to die than cry” – where tolerance of pain and discomfort is seen as a positive attribute of masculi-nity. It is plausible (but unproven) that men are further along in their COVID-19 infection by the time they seek medical care, and therefore more likely to require hospital admission.

While men may be seeking care later in the course of their COVID-19 infection, conversely, gendered systems and structures may be reducing women’s access to and use of hospital-based care for a range of conditions including COVID-19. Evidence from a range of countries shows a consistent pattern of lower health care use among poorer women, women who are more geographically isolated and those with less access to financial resources including health insurance – a position that is exacerbated by the concurrent findings that such women are more likely to suffer catastrophic health expenditures when they do access health care (Brinda, Andrés, and Enemark 2014; Onah and Govender 2014; Amaya-Lara 2016; Saikia Nandita and Bora 2016). Once women are in the health system, there is a large body of evidence highlighting the lower quality of overall care they are likely to receive for a variety of conditions – including cardiac care (Radovanovic et al. 2007; Shaw et al. 2017).

Again, it is plausible that women may be less likely to access hospital-based care in many settings, particularly where there is no financial support for their use of health services. Such inequalities in service access may underlie the differences seen in the global and national data showing lower rates of COVID-related hospital admissions among women. Evidence for this hypothesis is, however, currently lacking – but highlights the need not only to have more research on pathways of care in COVID-19, but also to ensure that such research takes gender into account.

COVID-19 is also impacting on pathways of care for other health conditions – and may be contributing to gender inequalities as a result. For example, the implications of the pandemic on sexual and reproductive health and rights (SRHR) have been devastating in some settings, particularly on women (who, as noted above, use SRH services more frequently than men do). As health systems struggle to deal with the additional workload as a result of people suffering from COVID-19, it is predicted that core existing services are being cut back due to resource diversion and the impact of the epidemic on the health workforce itself (Chattu and Yaya 2020). Staff from one of the largest global providers of SRH services report, for example, that clinics have closed or are operating on reduced hours, mobile outreach services have been suspended and supply chains disrupted. As a result, there have been “declining client numbers across all channels” (Church, Gassner, and Elliott 2020, 1). Sadly, this follows the same pattern as seen in previous epidemics such as Ebola in West Africa. In Sierra Leone, for example, during the 2014–2015 Ebola virus epidemic, it has been estimated that only a quarter of women who required Caesarian sections received them (Ribacke et al. 2016); women’s lack of access to antenatal and obstetric care saw significant increases in rates of both maternal mortality and stillbirths during the epidemic (Jones et al. 2016).

### Gender, COVID-19 and risk of death

The risk of death among people infected with COVID-19 is higher among people with pre-existing co-morbidities (i.e. other diseases present) than among those without any underlying illness. From the earliest reports of the pandemic in China and Italy, studies have highlighted the association between the presence of an underlying health problem and risk of death (COVID-19 Surveillance Group 2020; Guan et al. 2020). In particular, co-morbidities classified as “non-communicable diseases” (NCDs) appear to carry a particular risk of severe disease (e.g. requiring admission to intensive care) and death.

The NCDs are a wide-ranging category in global health, but the most common conditions include diabetes, cancers, heart disease, lung disease and strokes. In 2018 NCDs accounted for over 70 per cent of all deaths (NCD Countdown 2018), a large proportion of which is premature mortality (below the age of 70 years). NCDs are not evenly distributed across society. Poverty, socio-economic status, gender and education are all associated with higher rates (Barbeau, Leavy-Sperounis, and Blabach 2004; Cortese and Ling 2011; Williams et al. 2018), in part driven by higher rates of exposure to risks (see below), but also by a reduced capacity to protect health. For example, a systematic review of NCD risks in low- and middle-income countries found that less affluent groups “consume the least healthy diet” (Allen et al. 2017).

An individual’s risk of developing an NCD is driven by a range of exposures across the lifetime – foremost of which are diet, smoking tobacco, drinking alcohol and exposure to air pollution. Each of these risk factors is strongly associated with a risk of developing NCDs but is also highly gendered. Take the case of tobacco: smoking tobacco is ranked among the top five causes of (avoidable) disability and premature mortality globally (Reitsma et al. 2017), and a meta-analysis of risk of severe COVID-19 disease (including likelihood of death) confirmed that people with a history of smoking were almost two times more likely to develop severe disease compared to never smokers (Patanavanich and Glantz 2020).

Smoking, like many risk exposures, is a gendered behaviour. Sex-disaggregated smoking prevalence data from all regions of the world show that globally around 83 per cent of younger (age 15–24 years) smokers and 86 per cent of smokers aged 25–69 years are men (WHO 2018). These rates reflect decades of activity by the tobacco industry to construct and benefit from the exploitation of gender norms in relation to smoking. Evidence from industry archives, for example, show that in the 1950s, the advertising company Leo Burnett launched a campaign linking smoking Marlboro cigarettes to cowboy imagery on the grounds that cowboys are “an almost universal symbol of admired masculinity” (Burnett 1955). Through intense industry efforts, smoking became seen as a positive aspect of masculinity (Courtenay 2000) including the concept of “masculine risk-taking” (Wilsnack, Wilsnack, and Obot 2005).

Gender is not immutable, and the tobacco industry has both recognised and exploited the flexibility of gender norms with the aim of reaching untapped potential markets – i.e. women. In the higher income economies of Europe and North America, women have been targeted by an industry intent on portraying tobacco smoking as equated with positive notions of sexuality, weight control and independence (Brandt 1996; Amos and Haglund 2000; Hu and Lee 2016). Smoking rates among women in these areas are more than twice as high as the global average. Moreover, the tobacco industry has set its sights on the large non-smoking population of women in Asia and Africa and is actively pursuing markets opportunities in these regions (Gilmore et al. 2015).

Tobacco is illustrative of a wide range of gendered exposures that increase lifetime risks associated with developing the NCDs which have proven to be strongly associated with a severe outcome in COVID-19. Alcohol use and poor diet (diet high in processed foods, low in fresh grains, nuts, fruits, vegetables) are also frequently more common among men than women in many regions, and contribute to the higher burdens of common NCDs seen in men in most countries (WHO 2019b). Moreover, country-level analysis of COVID-19 deaths and women’s workforce participation finds a positive association between these two variables (Adams 2020) – lending credence to the hypothesis that it is not just individual gendered behaviours that drive risk of NCDs/COVID-19, but environmental exposures including through occupations too.

## Discussion

The COVID-19 pandemic is not gender-neutral. From the risk of infection to the risk of death, the distribution of the epidemic across communities and societies illustrates the underlying social and structural drivers of ill-health more widely and reflects the gendered nature of these drivers more specifically. As we have seen in the evidence presented throughout this paper, gender – embodied, enacted and embedded in behaviours, structures, systems and intersecting with other markers of division within societies – directly influences the distribution of risk associated with a global pandemic.

This is not an unexpected finding. The history of gender within global public health has consistently illustrated the importance of this social construction in driving not only life chances but life expectancy and rates of health and wellbeing (Hawkes and Buse 2020a). Concurrently, despite evidence of profound influence, gender is only infrequently taken into account when designing and implementing public health policies,programmes or practices that seek to respond to the risks and impact of most diseases (Hawkes and Buse 2013). COVID-19 is no exception.

For some aspects of COVID-19, the gendered impact of the epidemic has been taken into consideration – in particular in relation to the social and economic impacts on women/girls and the potential of the epidemic to reverse recent progress towards gender equality (UNWOMEN and UN Secretary General 2020). This has drawn some benefits: the likelihood that domestic violence rates would surge during lockdowns was considered in pandemic preparedness plans in some countries, and additional financial resources were allocated to support women’s refuges and other forms of social, legal and justice-based support (e.g. see plans from Government of Canada 2020). Gender-responsive planning has, however, been patchy and inconsistent, and globally the world has witnessed what the UN Secretary-General has called a “horrifying surge in domestic violence” (Guterres 2020b). Moreover, although there is evidence that economic hardships – including job losses – are more likely to affect women, only a small number of countries have taken gender into account in the design and delivery of social protection schemes addressing the disastrous economic impacts of COVID-19 on house-holds, communities and societies (Wenham et al. 2020). In other words, the gendered impact of COVID-19 on the lives and livelihoods of all people is clear – even if the gender-responsive policies and programmes to redress inequalities and inequities are frequently afterthoughts, inadequate and/or under-resourced.

Where gender has featured in the discussion on risk exposure, it has often been based on limited evidence and has given salience to individual responsibility (or lack thereof) as opposed to deeper structural gendered relations. For example, initially there was much media hand-wringing over men’s reported poor personal hygiene and specifically their reported lower rates of hand washing (see e.g. Krueger 2020). The notion that poor hand hygiene may be linked to the lack of public rest rooms accessible to those working in the predominantly male transport and delivery industries, overly short breaks for workers in industrial settings and a range of other potential structural impedi-ments, receives far less media coverage. This framing reflects an ongoing neoliberal ideo-logical focus on the individual as opposed to the structural impediments to mitigating risk in global health (Yang, Mamudu, and John 2016; Purdie, Buse, and Hawkes 2019). While COVID-19 has reinvigorated and widened the range of voices calling for universal labour guarantee, wealth taxes, publicly provided health care and a state capable of regulating markets (Ahmed 2020), meanwhile the gender debate seems to be stuck at the level of gender clichés as evidenced for example in whether the wearing of protective masks is “emasculating” (Glick 2020).

While some gender-related issues have surfaced, and lip service paid or action taken, other issues have had less airtime or not been raised in mainstream public health at all. Often these “overlooked” or “neglected” issues have been about who stands to gain from the crisis, whose interests are reinforced, which ideas are privileged at the expense of others, and similarly which institutions are seen to be needed and which to be challenged. Attention to some of these issues over others is a reflection of deep structural elements in which gender is bound up.

The gender-blindness of public health responses may not have been unexpected, but it carries with it a risk of overlooking the deep structural and social drivers of population ill-health and vulnerability to current and future pandemics. WHO’s COVID-19 Strategy, for example, only mentions gender twice – both times in relation to women/ girls and risks of gender-based violence and the need for “safeguarding” (WHO 2020). Six months into the pandemic, our global data-tracker finds that only a minority of the world’s countries fully report COVID data disaggregated by sex – meaning that any opportunity for understanding the distribution of infection, disease and patterns of healthcare use is lost, and our ongoing reviews of policies and programmes in COVID-19 control find a stark absence of gender-responsiveness (Global Health 50/50 COVID-19 data tracker).

Applying a gender lens to understanding the drivers of ill-health in relation to COVID-19 reveals the deeply ingrained role played by structural and commercial determinants of health driving differences in sex-disaggregated outcomes. In terms of exposure to the virus, gendered divisions intersect with occupation and migration to drive inequities. When health and social care, particularly in the lower paid centiles of these occupations, are seen as “female” occupations, this is reflected in national data which find that the majority of infected health workers are women. In some settings in high-income countries, inequalities in risk of exposure may be further reinforced through the intersection of gender with other structural drivers such as ethnicity and migration status (e.g. in the UK 8% of health workers are from non-EU countries, mainly South Asia and the Philippines – ONS 2019). Likewise, when the economy of both country and family is unsustainable except through the export of labour, the prevailing expectation in many societies is that men should fulfil this role by migrating and sending home remittances. As noted, the overcrowded living conditions experienced by these men have likely contributed to their over-representation in the male-heavy distribution of infections in both “receiving” and “sending” economies.

The widest disparities are seen in the risk of death. Although case fatality rates – i.e. number of deaths among number of cases – are fraught with potential for bias in calculation and interpretation (Kenyon 2020), nonetheless these rates provide an opportunity to compare rates in men and women using the same methodological approach. Data presented by the Global Health 50/50 COVID-19 data tracker find that an overwhelming majority of countries report a higher risk of death among men compared to women. Biological sex is playing a role, but gender is driving a significant proportion of this mortality difference.

The chronic NCDs that appear to be associated with the higher COVID-19 risk of death are driven and sustained by commercial and other structural determinants. These determinants serve to produce unhealthy living and working environments where, for people and populations, the realisation of the right to health is frequently determined (negatively) by the activities of powerful organisations – most often in the private for-profit sector. Thus, there is a rich body of literature examining the activities, including the political activities (Mialon et al. 2016), of transnational (and national) corporations in relation to health outcomes (see, for example, Stuckler et al. 2012; Moodie et al. 2013; Baum et al. 2016). A large part of the literature highlights that many activities of the private sector frequently escape sufficient regulation or oversight owing to the lack of state capacity to promote and sustain healthy environments – including in relation to food/diet, air quality and exposure to health-harming products such as alcohol and tobacco (Buse, Tanaka, and Hawkes 2017).

Evidence on health inequities shows that men currently appear to be at higher risk in these unregulated, harmful environments in many parts of the world. When ill-health is driven, in part, by the unregulated impact of the (capitalism-based) market (Sell and Williams 2019), there appears to be an ongoing relationship between the power (including the resource-based power) to participate in the market, and the risk of ill-health. Thus, when men are the ones who, over decades, have had the purchasing power to consume unhealthy products (tobacco, alcohol, ultra-processed diets), this will be reflected in ill-health statistics that show higher rates of exposure-related illnesses in men compared to women. For example, an analysis of cohort data in high-income countries by Beltrán-Sánchez, Finch, and Crimmins (2015) found that over a period of more than 100 years, 30 per cent of excess male mortality was calculated as being attributable to smoking tobacco – with its consequent impact on risk of heart disease, stroke and cancers.

Similarly, when the market relies on the availability and mobility of cheap labour, and norms of masculinity are entwined with gendered constructions of who is permitted in the formal or informal employment sector (ILO 2018), or who is expected/allowed to travel (men), then men are at higher risk of the negative consequences associated with the poor living (and working) conditions suffered by many labour migrants and hence exposure to COVID-19.

None of this analysis serves to detract from the ongoing inequalities and injustices suffered by women and girls in the COVID-19 pandemic. Rates of insecure employment, job losses, gender-based violence – all sadly serve to illustrate the impact of gender inequalities suffered by women globally. However, by applying a more comprehensive and nuanced gender lens to the COVID-19 pandemic, we have shown that the negative impact of the pandemic is widespread across society and not confined to one half of the population alone.

Taking a gender lens to analysing the impact of COVID-19 is the first step towards more gender-responsive policies, programmes and practices – an approach that will, ultimately, benefit the health of all people not only in the case of this current pandemic but for improving health more holistically and equitably for all.

The evidence suggests that moving toward such policies is more likely to happen where more gender-equal governance structures are in place. The correlation between women’s leadership of 10 countries (Belgium, Denmark, Estonia, Finland, Germany, Greece, Iceland, New Zealand, Norway and Taiwan) and relatively more effective COVID-19 policies and better outcomes has been noted. These are countries, it is argued, that already had “a stronger focus on social equality, human needs and generosity” and whose citizens were “receptive to political agendas that place social and environmental wellbeing at the core of policymaking” (Coscieme et al. 2020). Thus, the putative benefits of women’s leadership need to be seen in a societal context. Coscieme et al. (2020) further note that “women to be more likely to take up positions of political leadership in societies that value equity, solidarity, nurturing, and collaboration, which are usually associated with healthier communities”. At the level of institutions, an analysis of 200 global health organisations found that more gender-equitable leadership in those organisations was correlated with greater concern for gender equality and diversity in those organisations (Global Health 50/ 50 COVID-19 data tracker).

Arundhati Roy, the novelist and activist, described COVID-19 as “a portal, a gateway between one world and the next” and that we can choose what world we want to emerge into on the other side (Roy 2020). The evidence suggests that intersectional feminist values of equality, fairness and social justice would serve to protect people from pandemics, and that is our vision and the struggle we are part of as we step through the portal.
